# Admission Serum Bicarbonate Predicts Adverse Clinical Outcomes in Hospitalized Cirrhotic Patients

**DOI:** 10.1155/2021/9915055

**Published:** 2021-05-17

**Authors:** Michael Schopis, Anand Kumar, Michael Parides, Adam Tepler, Samuel Sigal

**Affiliations:** ^1^Department of Medicine, Montefiore Medical Center, Albert Einstein College of Medicine, Bronx, NY 10467, USA; ^2^Division of Gastroenterology, Montefiore Medical Center, Albert Einstein College of Medicine, Bronx, NY 10467, USA; ^3^Division of Gastroenterology, Lenox Hill Hospital, New York, NY 10075, USA; ^4^Department of Surgery, Montefiore Medical Center, Albert Einstein College of Medicine, Bronx, NY 10467, USA; ^5^Division of Hepatology, Montefiore Medical Center, Albert Einstein College of Medicine, Bronx, NY 10467, USA

## Abstract

A low serum bicarbonate (SB) level is predictive of adverse outcomes in kidney injury, infection, and aging. Because the liver plays an important role in acid-base homeostasis and lactic acid metabolism, we speculated that such a relationship would exist for patients with cirrhosis. To assess the prognostic value of admission SB on adverse hospital outcomes, clinical characteristics were extracted and analyzed from a large electronic health record system. Patients were categorized based on admission SB (mEq/L) into 7 groups based on the reference range (22–25) into mildly (18–21), moderately (14–17), and severely (<14) decreased groups and mildly (26–29), moderately (30–33), and severely (>30) increased groups, and the relationship of SB category with the frequency of complications (acute kidney injury/hepatorenal syndrome, portosystemic encephalopathy, gastrointestinal bleeding, ascites, and spontaneous bacterial peritonitis) and hospital metrics (length of stay [LOS], admission to an intensive care unit [ICU], and mortality) was assessed. A total of 2,693 patients were analyzed. Mean SB was 22.9 ± 4.5 mEq/L. SB was within the normal range (22–25 mEq/L) in 1,072 (39.8%) patients, and 955 patients (36%) had a low SB. As the SB category decreased, the incidence of complications progressively increased (*p* < 0.001). Increased MELD-Na score and low serum albumin also correlated with frequency of complications (*p* < 0.001). As the SB category decreased, LOS, ICU admission, and mortality progressively increased (*p* < 0.001). On multivariate analysis, the association of decreased SB with higher odds of complications, LOS, ICU admission, and mortality persisted. *Conclusion*. Low admission SB in patients with cirrhosis is associated with cirrhotic complications, longer LOS, increased ICU admissions, and increased hospital mortality.

## 1. Introduction

Acid-base disturbances are common in patients with cirrhosis. In early stages of cirrhosis, acidosis results from dilutional hypervolemia and hyperchloremia, whereas alkalosis occurs due to hypoalbuminemia and respiratory alkalosis [1]. As a result, many cirrhotic patients demonstrate a complex combination of acidosis and alkalosis [1, 2]. As the severity of cirrhosis progresses, patients often develop a net metabolic acidosis, especially in those with acute and chronic liver failure with sepsis in which increased levels of lactic acid and unmeasured anions accumulate [1, 3, 4]. The resulting acidosis is frequently accompanied by a decreased serum bicarbonate (SB) level [3].

Acid-base imbalances with decreased SB levels are also common in patients with chronic kidney disease (CKD), acute kidney injury (AKI), and infection, and the elderly. In AKI and CKD low SB is associated with increased severity of illness and is predictive of adverse hospital outcomes and mortality [5, 6]. Additionally, low SB correlates with increased hospital length of stay (LOS) in patients with cellulitis and with increased mortality in the elderly and in trauma intensive care unit (ICU) admissions [7–9].

The significance of SB in cirrhosis has only received limited attention. Most studies have assessed the impact of increased serum lactate levels and unmeasured anion acidosis on ICU mortality [3, 4, 10–12], whereas only three studies have evaluated SB as a prognostic marker [4, 13, 14]. Based on the significance of acidosis in cirrhosis and low SB in other chronic disease states, we speculated that SB would be predictive of adverse hospital outcomes for the hospitalized cirrhotic patient.

## 2. Materials and Methods

### 2.1. Subjects

A retrospective cohort study was conducted on data extracted from an electronic health record system (EPIC®) using Clinical Looking Glass© (Emerging Health Information Technology, Yonkers, NY). EPIC is a computerized patient database that contains comprehensive data, including patient demographics, hospitalizations, discharge diagnoses (International Classification of Diseases [ICD] codes), laboratory and imaging results, histopathology, endoscopic and surgical procedures, and medications. Clinical Looking Glass© is a proprietary software that permits exploration of the database contained within EPIC®. Using Clinical Looking Glass©, it is possible to obtain desired clinical data on consecutive patients meeting predefined criteria. The study was approved by the Institutional Review Board at the Albert Einstein College of Medicine.

Diagnoses were based on ICD, 9th revision or 10th revision, clinical modification codes recorded at hospital discharge. Patients analyzed included those aged ≥18 years with a diagnosis of cirrhosis (ICD-9 : 571.2, 571.5, 571.6, 571.42, 571.49, 571.5, 571.6, 571.8; ICD-10 : K70.30, K70.31, K74.60, K74.69, K74.3, K74.4, K74.5, K75.4) between November 2015 and March 2019. Patients were excluded if a SB within 24 hours of admission was not available or if there was a discharge diagnosis of diabetic ketoacidosis (ICD-9 : 250.1; ICD-10 : E10.10), acute coronary syndrome (ICD-9 : 410, 411; ICD-10 : I21, I22, I23, I24), or fulminant liver failure (ICD-9 : 570; ICD-10 : K72.00). Patients were also excluded if there was a preexisting diagnosis of end-stage renal disease (ICD-9 : 585.6; ICD-10 : N18.6), chronic obstructive pulmonary disease (ICD-9 : 491, 492, 494, 496; ICD-10 : J40, J41, J42, J43, J44, J47), systolic heart failure (ICD-9 : 428.2; ICD-10 : I50.2), or organ (liver, kidney, heart) transplantation (ICD-9 : V42.0, V42.1, V42.6, V42.7, V42.83, V42.84; ICD-10 : Z94.0, Z94.1, Z94.2, Z94.4, Z94.82, Z94.83) as well as a prescription for bicarbonate therapy prior to hospitalization. For patients with more than one hospitalization during the time period, only the index admission was analyzed.

### 2.2. Admission SB Stratification and Clinical Characteristics

Patients were categorized by admission SB milliequivalents per liter (mEq/L) into 7 groups based on the reference range (22–25) that spanned 4 mEq/L into those with mildly (18–21), moderately (14–17), and severely (<14) decreased levels and mildly (26–29), moderately (30–33), and severely (>34) increased levels as previously described in studies examining prognosis of SB on mortality and cardiovascular events in kidney transplant recipients [15]. Admission clinical characteristics for the entire cohort and for each SB category were recorded.

### 2.3. Relationship of Admission SB Category and Adverse Hospital Metrics

Patient discharge diagnoses were queried for complications of renal failure (AKI/hepatorenal syndrome [HRS], ICD-9 : 584, 572.4; ICD-10 : N17, K76.7), portosystemic encephalopathy (PSE, ICD-9 : 572.2; ICD-10 : 1K70.41, K72.11, K72.91), gastrointestinal or variceal bleeding (GIB, ICD-9: 456.0, 456.2, 569.3, 578, 578.0, 578.9; ICD-10 : I85.01, I85.11, K92.0, K92.1), ascites (ICD-9 : 789.5; ICD-10 : K70.31, R18.8), and spontaneous bacterial peritonitis (SBP, ICD-9: 567.23; ICD-10 : K65.2). The percentage of patients with each complication in the various SB categories was recorded.

### 2.4. Relationship of Admission SB Category and Hospital Metrics

Patient discharge records were queried for LOS, requirement for ICU care, and mortality.

Average LOS and the percentages of patients who required ICU admission and died during the hospitalization were determined for each SB category.

### 2.5. Statistical Analysis

Summary statistics were described as means and standard deviations or counts and percentages. Categorical variables between SB groups were compared using Pearson's chi-squared test or Fisher's exact test, and continuous variables were compared using the analysis of variance (ANOVA). General linear model was used to examine the difference in LOS between the SB groups adjusting for covariates. Multivariable logistic regression models were used to calculate adjusted odds ratios (aOR) of cirrhosis-related complications, ICU admission, and mortality. All models were adjusted for clinically significant covariates (i.e., age, gender, Model of End-Stage Liver Disease-Sodium [MELD-Na], and serum albumin [SA]). Amongst the 7 SB groups, SB 22–25 was considered as the reference group against which other SB groups were compared. Statistical significance was set at *p*-value <0.05. All analyses were performed using IBM SPSS, Version 25.0 (IBM Corp., Armonk, NY).

## 3. Results

### 3.1. Subjects

Between November 2015 and March 2019, 3,663 patients with cirrhosis aged ≥18 years were hospitalized, of which 3,540 had a SB level measured within the first 24 hours of admission.

A total of 2,693 admissions were available for analysis after excluding patients with diabetic ketoacidosis (*n* = 134), acute coronary syndrome (*n* = 21), fulminant liver failure (*n* = 54), end-stage renal disease (*n* = 296), chronic obstructive pulmonary disease (*n* = 129), systolic heart failure (*n* = 421), previous transplantation (*n* = 206), and preadmission supplemental bicarbonate therapy (*n* = 128).

### 3.2. Admission SB Stratification and Clinical Characteristics

Baseline characteristics of the study cohort and the various SB groups are presented in Table 1. Mean SB was 22.9 ± 4.5 mEq/L. SB was within the normal range (22–25 mEq/L) in 1,072 (39.8%) patients, and 955 patients (36%) had a SB below the reference range. Sixty-six patients had severely decreased SB (<14 mEq/L), 227 moderately decreased SB (14–17 mEq/L), 662 mildly decreased SB (18–21 mEq/L), 531 mildly increased SB (26–29 mEq/L), 100 moderately increased SB (30–33 mEq/L), and 35 severely increased SB (>30 mEq/L). Mean age was 61 ± 12 years, and 61% were male. Recorded causes of cirrhosis were alcohol (37.2%), hepatitis C virus (33.3%), nonalcoholic steatohepatitis/cryptogenic (21.7%), hepatitis B virus (3.6%), and other (autoimmune hepatitis, primary biliary cirrhosis, primary sclerosing cholangitis; 4.1%).

Age and gender distributions were similar across the SB groups. Patients with lower SB had more advanced liver disease. Serum creatinine, alanine aminotransferase, alkaline phosphatase, total bilirubin, international normalized ratio, and MELD-Na were higher in the lower SB groups, while SA was lower (*p* < 0.05).

### 3.3. Relationship of Admission SB Category and Cirrhosis Complications

The frequency of cirrhosis complications and adverse hospital metrics among SB categories are presented in Table 2. Univariate associations of SB categories with cirrhosis complications are presented in Table 3. As the SB category decreased compared to the reference range, the incidence of renal failure, PSE, GIB, ascites, and SBP progressively increased (*p* < 0.001). Even a mild decrease in SB (18–21) from reference range resulted in a marked increase in frequency of SBP and renal failure [OR: 2.44 (95% CI: 1.5–3.95) and 2.36 (1.87–2.98), resp.]. Additional factors predictive of complications on univariate analysis included age, gender, MELD-Na, and SA (*p* < 0.05). Older age was associated with renal failure, and younger age and a male gender were associated with ascites (*p* < 0.05). MELD-Na score correlated with renal failure, PSE, and ascites. Lower SA was associated with renal failure, PSE, GIB, ascites, and SBP (*p* < 0.05).

The factors predictive of cirrhosis complications on multivariate analysis are presented in Table 4. SB <14 was independently associated with a higher odds of a diagnosis of AKI/HRS [aOR 8.49 (4.31–16.72)], SBP [aOR 2.1 (1.01–4.52)], PSE [aOR 2.22 (1.08–4.56)], and GIB [aOR 2.19 (1.12–4.27)]. SB 14–17 predicted higher odds of AKI/HRS [aOR 3.64 (2.53–5.24)], PSE [aOR 1.69 (1.03–2.76)], and GIB [aOR 1.56 (1.02–2.40)]. In contrast, SB higher than the reference range (SB 26–29) was associated with a lower odds of GIB [aOR 0.40 (0.25–0.65)]. Additional factors on multivariable analysis predictive of cirrhosis complications included lower SA (all complications) and higher MELD-Na (AKI/HRS, PSE, ascites, and SBP).

### 3.4. Relationship of Admission SB Category and Adverse Hospital Metrics

#### 3.4.1. Hospital LOS

Mean LOS was 9 ± 11 days. As the SB category decreased compared to the reference range, LOS progressively increased (Table 5; *p* < 0.001). Even a mild decrease in SB (18–21) from reference range resulted in a substantial increase in LOS. Additional factors associated with increased LOS on univariate analysis included increased MELD-Na and lower SA. Older age, in contrast, was associated with shorter hospital LOS (*p* < 0.001).

Results of multivariate analysis of the factors associated with adverse hospital metrics are presented in Table 6. Patients in SB categories <14, 14–17, and 18–21 had significantly longer hospital LOS compared to those with SB within the reference range after adjusting for covariates [mean difference 4.07 (1.21–6.93), 3.14 (1.48–4.81), and 1.46 (0.31–2.6) days, resp.]. Higher MELD-Na and lower SA also predicted a longer LOS (0.2 days per unit increase and 1.5 days per unit decrease, resp.), and older age predicted a shorter LOS (−0.04 days per increase, *p*=0.02).

#### 3.4.2. ICU Admission

A total of 233 (8.7%) patients required ICU care. The odds of ICU admission among the SB groups on univariate analysis are presented in Table 5. Low SB was associated with ICU admission. Additional risk factors for ICU admission included older age, higher MELD-Na, and lower SA (*p* < 0.05). On multivariate analysis (Table 6) SB <14, 14–17, and 18–21 remained independent predictors of ICU admission [aOR: 6.5 (3.5–11.9), 2.4 (1.5–3.8), and 1.7 (1.20–2.46), resp.]. Other significant risk factors for ICU admission included older age [aOR 1.7 (1.04–2.87)] and lower SA [aOR 0.67 (0.6–0.80)].

#### 3.4.3. Hospital Mortality

172 (6.4%) patients died during the hospitalization. The odds of hospital mortality among the SB groups on univariate analysis are presented in Table 4. Declining SB, older age, higher MELD-Na, and lower SA were all associated with mortality. On multivariate analysis (Table 6) SB 14–17 predicted hospital mortality [aOR 1.7 (1.04–2.87)]. Other significant risk factors for mortality included older age [aOR 1.04 (1.02–1.05)], higher MELD-Na [aOR 1.11 (1.08–1.13)], and lower SA [aOR 0.50 (0.38–0.65)].

## 4. Discussion

In this study we report that admission SB was an important prognostic marker for adverse hospital outcomes for the cirrhotic patient. Low admission SB was significantly associated with an increased risk of a discharge diagnosis of renal failure, PSE, GIB, and SBP. In addition, low SB was significantly associated with longer hospital LOS, ICU admission, and inpatient mortality. Although higher MELD-Na had a similar correlation [16, 17] and higher admission albumin had a protective effect [18] as has been previously reported, the impact of admission SB persisted after adjusting for these variables. The finding of lower LOS among older patients was unexpected but might be explained by a higher mortality rate.

The liver performs a variety of metabolic processes involved in acid-base homeostasis. These include acidifying processes such as urea production and synthesis of albumin and ketoacids and alkalizing ones such as metabolism of lactate and amino acids. [1] Importantly, the healthy liver is responsible for the metabolism of up to 70% of all serum lactate with its conversion to serum bicarbonate via the Cori Cycle [19].

Patients with cirrhosis of increasing severity have progressively impaired acid-base regulation [1]. Compensated hypocapnic respiratory alkalosis is common in stable early cirrhosis [1]. In advanced cirrhosis, portal hypertension-induced vasodilation leads to low effective circulatory volume and subsequent upregulation of compensatory mechanisms that, in turn, lead to increased resorption of free water and resultant dilutional acidemia [20, 21]. Additional factors that affect acid-base status include activation of the renin-angiotensin-aldosterone system, diarrhea, and diuretic use. There is also accumulation of unmeasured anions attributed to uremic acidosis and ketoacidosis from dysregulated ketogenesis [1, 21] and reduced hepatic amino acid uptake [1, 22].

Net acidosis is frequently encountered in the cirrhotic patient with acute on chronic liver failure and sepsis that is closely associated with hyperlactacidemia [1]. Lactate is a marker of tissue hypoxia due to impaired mitochondrial oxidation [23]. Patients with decompensated cirrhosis have increased lactate production due to tissue malperfusion, impaired cellular oxygen metabolism, and a hypermetabolic state as well as reduced lactate clearance by the cirrhotic liver [19, 24, 25]. All of these acidifying factors are only moderately balanced by the alkalizing effect of hypoalbuminemia and tachypnea [26, 27].

The importance of acidosis in cirrhosis has been most extensively studied in relation to elevated lactate levels in the ICU setting. In a retrospective study comparing the acid-base profile of 178 patients with acute on chronic liver disease to that of 178 patients without liver disease, the lactate level on admission to the ICU predicted mortality only in patients with liver disease [4]. The prognostic value of lactate levels during ICU admission in liver disease was validated in a separate cohort in which it was directly associated with vasopressor use, bilirubin and INR levels, Acute-on-Chronic Liver Failure (ACLF) grade, and 28-day mortality and 1-year mortality [11].

There are multiple potential pathophysiologic processes that lead to a low SB level in the cirrhotic patient. In early cirrhosis compensatory renal acidification via decreased excretion of tubular hydrogen ions and ammonium and increased bicarbonate excretion balance the alkalizing effects of hypoalbuminemia and chronic respiratory alkalosis. A new steady state develops in which the kidney chronically suppresses bicarbonate reabsorption in return for increased chloride reabsorption, leading to low SB [28–30]. Diarrhea, which frequently occurs with lactulose therapy, is associated with the gastrointestinal loss of bicarbonate [31]. Patients with fatty liver disease often have concurrent insulin resistance which has been associated with acidosis and low SB due to excess ketone bodies [32]. Elevated intrarenal ammonia levels activate chemotactic and cytolytic complement components leading to tubule-interstitial inflammation and acidosis [33]. The effect of metabolic acidosis on renal tissue and decreased SB is further exacerbated by greater in situ activation of angiotensin II, aldosterone, and endothelin [34–36]. Spironolactone therapy is associated with serum potassium inhibition of ammonia production and subsequent metabolic acidosis [37]. In the hospitalized cirrhotic patient, administration of large volume saline can lead to hyperchloremic acidosis [1]. Finally, there is impaired retransformation of lactate to glucose and an equimolar release of bicarbonate in the liver by the Cori Cycle in critically ill patients [38].

SB level decreases in a linear fashion with increasing acid load [39], and a low SB predicts the presence of significant metabolic acidosis more reliably than pH, the anion gap, and the lactate level [8]. However, the clinical significance of SB levels in cirrhosis has received relatively little attention. In a study of 178 ICU patients with cirrhosis, those with ACLF had significantly lower SB levels than the cirrhotic patients without ACLF (18.9 mEq/L versus 22.7 mEq/L), and a low SB level was associated with 28-day mortality. [4] In a follow-up study of 185 cirrhotic patients admitted to the ICU, the SB had prognostic significance for 7-day mortality [14]. Finally, the SB level on admission was an independent predictor of ICU mortality in 177 critically ill patients with cirrhosis, and replacement of the bilirubin level with the SB level to create a “MELD-bicarbonate” score actually outperformed the original MELD score in predicting mortality [13].

Our study is the first to examine the prognostic significance of admission SB among all hospitalized cirrhotic patients with respect to adverse hospital outcomes. The attractiveness of the use of SB as a potential prognostic marker is that it is readily available as a standard test for all patients in all hospitals without the requirement for special preparation or testing, and it provides an indirect estimate of total acid accumulation. A strength of the study was the use of a large sample size and a diverse patient population. Limitations included the retrospective nature of the analysis and the use of discharge ICD coding which cannot distinguish diagnoses present on admission from those that develop during the hospitalization and the use of a one-time SB assessment. Future studies are necessary to validate our findings and to determine a possible relationship between changes in SB levels and outcomes.

## Figures and Tables

**Table 1:** Baseline characteristics of study cohort (*N* = 2693).

Baseline characteristic	Mean ± SD	SB < 14 (*N* = 66, 2.5%)	SB 14–17 (*N* = 227, 8.4%)	SB 18–21 (*N* = 662, 24.6%)	SB 22–25 (*N* = 1072, 39.8%)	SB 26–29 (*N* = 531, 19.7%)	SB 30–33 (*N* = 100, 3.7%)	SB >33 (*N* = 35, 1.3%)
Age^§^	61.1 ± 12.3	60.5 ± 13.4	60 ± 13	60.7 ± 12.5	61.3 ± 12.4	61.8 ± 11.5	62 ± 13	61.1 ± 11.3
Male/female	1642/1051	46/20	138/89	406/256	636/436	335/196	59/41	22/13
Initial SB^§†^	22.9 ± 4.5	10.4 ± 2.7	16.0 ± 1.0	19.9 ± 1.1	23.5 ± 1.1	27.1 ± 1.1	31.1 ± 1.1	36.5 ± 3.4
Initial pH^†^	7.38 ± 0.08	7.21 ± 0.17	7.33 ± 0.09	7.38 ± 0.07	7.39 ± 0.05	7.38 ± 0.05	7.39 ± 0.07	7.38 ± 0.09
Initial BUN^§†^	91.3 ± 239.2	162.7 ± 319.2	105.5 ± 108.7	105.4 ± 424.6	82.0 ± 95.3	77.6 ± 142.7	92.3 ± 180.3	68.0 ± 67.7
Initial Cr^§†^	1.19 ± 1.04	2.76 ± 2.95	2.04 ± 1.95	1.29 ± 0.95	0.99 ± 0.52	0.96 ± 0.50	1.02 ± 0.68	0.95 ± 0.53
Initial Cl^§†^	100.2 ± 6.1	101.0 ± 9.4	101.3 ± 7.8	101.1 ± 6.2	100.5 ± 5.5	99.0 ± 5.2	96.6 ± 6.0	92.8 ± 6.4
Initial CO_2_^§†^	42.7 ± 9.8	31.3 ± 13.0	34.9 ± 7.1	38.5 ± 6.3	42.6 ± 6.5	48.8 ± 8.1	54.2 ± 10.4	66.3 ± 20.1
Initial Na^§†^	137.8 ± 5.2	137.8 ± 6.9	136.2 ± 6.2	137.1 ± 5.4	138.0 ± 4.8	138.5 ± 4.5	138.5 ± 5.1	138.7 ± 5.3
AST^§^	91.7 ± 239.4	163.2 ± 319.2	105.5 ± 108.6	105.5 ± 423.9	82.9 ± 98.4	77.9 ± 142.3	92.2 ± 180.2	67.0 ± 67.7
ALT^§†^	49.8 ± 101.9	98.5 ± 355.9	54.0 ± 66.4	51.6 ± 106.6	46.2 ± 60.5	44.0 ± 80.3	67.2 ± 173.8	31.8 ± 24.1
Alk phos^§†^	155.6 ± 128.8	160.4 ± 122.5	176.4 ± 164.5	161.4 ± 132.8	152.8 ± 127.2	149.9 ± 117.0	139.2 ± 95.8	118.8 ± 58.5
Albumin^§†^	3.3 ± 0.7	3.0 ± 0.9	2.9 ± 0.8	3.2 ± 0.7	3.3 ± 0.7	3.4 ± 0.7	3.4 ± 0.7	3.2 ± 0.8
TB^§†^	3.0 ± 5.4	5.7 ± 10.2	5.3 ± 8.3	3.6 ± 6.0	2.6 ± 4.5	2.1 ± 3.3	1.8 ± 2.5	2.2 ± 3.8
INR^§†^	1.4 ± 0.8	1.9 ± 1.6	1.5 ± 0.7	1.4 ± 0.9	1.3 ± 0.5	1.3 ± 0.6	1.3 ± 0.5	2.0 ± 3.3
MELD^†^	13.8 ± 6.9	22.5 ± 9.8	18.6 ± 8.4	14.9 ± 6.9	12.6 ± 5.7	11.8 ± 5.7	11.8 ± 6.2	13 ± 5.9
MELD-Na^§†^	15.9 ± 8.3	23.8 ± 9.5	20.8 ± 8.5	17.1 ± 7.3	14.6 ± 7.3	13.5 ± 6.1	13.4 ± 7	14.6 ± 6.6

^§^Age (years at index date), SB units (mEq/L), BUN (mg/dL), Cr (mg/dL), Cl (mEq/L), CO2 (mEq/L), Na (mEq/L), AST (U/L), ALT (U/L), Alk Phos (U/L), albumin (g/dL), TB (mg/dL), INR ratio, and MELD-Na score. ^†^Significant difference in frequency between SB groups (*p* < 0.05).SD, standard deviation; N, sample size; SB, serum bicarbonate; BUN, blood urea nitrogen; Cr, creatinine; Cl, chloride; CO_2_, carbon dioxide; Na, sodium; AST, aspartate transaminase; ALT, alanine aminotransferase; Alk Phos, alkaline phosphatase; TB, total bilirubin; INR, international normalized ratio; MELD-Na, Model of End-Stage Liver Disease-Sodium.

**Table 2:** Frequency of outcomes among the study cohort^*∗*^.

Outcome	Total (*N* = 2693)	SB < 14 (*N* = 66)	SB 14–17 (*N* = 227)	SB 18–21 (*N* = 662)	SB 22–25 (*N* = 1072)	SB 26–29 (*N* = 531)	SB 30–33 (*N* = 100)	SB >33 (*N* = 35)
Renal failure (AKI/HRS)^†^	624 (23.2)	46 (69.7)	120 (52.9)	197 (29.8)	164 (15.3)	67 (12.6)	14 (14)	8 (22.9)
PSE^†^	193 (7.2)	12 (18.2)	32 (14.1)	47 (7.1)	60 (5.6)	27 (5.1)	9 (9)	4 (11.4)
GIB^†^	255 (9.5)	13 (19.7)	36 (15.9)	67 (10.1)	109 (10.2)	24 (4.5)	6 (6)	0 (0)
Ascites^†^	518 (19.2)	19 (28.8)	66 (29.1)	152 (23)	176 (16.4)	77 (14.5)	12 (12)	8 (22.9)
SBP^†^	173 (6.4)	10 (15.2)	27 (11.9)	51 (7.7)	55 (5.1)	21 (4.0)	6 (6)	3 (8.6)
ICU care^†^	233 (8.7)	22 (33.3)	36 (15.9)	69 (10.4)	69 (6.4)	26 (4.9)	8 (8)	3 (8.6)
Death^†^	172 (6.4)	11 (16.7)	37 (16.3)	49 (7.4)	50 (4.7)	17 (3.2)	7 (7)	1 (2.9)
Hospital LOS (days)^†^	8.8 ± 10.9	14.2 ± 15.8	12.8 ± 13.7	10 ± 12.4	7.6 ± 9.2	7 ± 8.4	8.9 ± 13.9	9.9 ± 11

^*∗*^Values represented as count (column %) or mean (±SD). ^^†^^Significant difference in frequency between SB groups (*p* < 0.001).SB, serum bicarbonate; AKI/HRS, acute kidney injury or hepatorenal syndrome; PSE, portosystemic encephalopathy; GIB, gastrointestinal bleed; SBP, spontaneous bacterial peritonitis; ICU, intensive care unit; LOS, length of stay.

**Table 3:** Univariable association of admission SB with cirrhosis complications during hospitalization.

Variable	AKI/HRS^†^	PSE^†^	GIB^†^	Ascites^†^	SBP^†^
SB 22–25^*∗*^	1	1	1	1	1
SB < 14	**13.22 (7.59–23.08)**	**3.75 (1.9–7.38)**	**2.17 (1.15–4.1)**	**2.06 (1.18–3.59)**	**4.96 (2.17–11.33)**
SB 14–17	**6.32 (4.64–8.60)**	**2.77 (1.76–4.37)**	**1.67 (1.11–2.5)**	**2.09 (1.5–2.9)**	**5.69 (3.35–9.65)**
SB 18–21	**2.36 (1.87–2.98)**	1.29 (0.87–1.91)	1 (0.72–1.37)	**1.52 (1.19–1.93)**	**2.44 (1.5–3.95)**
SB 26–29	0.77 (0.57–1.05)	0.9 (0.57–1.44)	**0.42 (0.27–0.66)**	0.86 (0.65–1.16)	0.41 (0.17–1.0)
SB 30–33	0.87 (0.48–1.57)	1.67 (0.8–3.47)	0.56 (0.24–1.32)	0.69 (0.37–1.3)	0.73 (0.17–3.12)
SB >33	1.58 (0.71–3.54)	2.18 (0.74–6.37)	—	1.51 (0.67–3.38)	**4.96 (2.17–11.33)**
Age^§^	**1.01 (1.004–1.02)**	1.01 (0.99–1.02)	**0.99 (0.98–0.999)**	**0.97 (0.96–0.98)**	0.99 (0.98–1.001)
Male vs female	1.16 (0.97–1.40)	1.10 (0.79–1.44)	0.92 (0.71–1.20)	**1.75 (1.42–2.16)**	1.02 (0.75–1.40)
MELD-Na^§^	**1.15 (1.13–1.16)**	**1.04 (1.03–1.06)**	1.01 (0.99–1.02)	**1.10 (1.08–1.11)**	**1.14 (1.12–1.17)**
Albumin^§^	**0.48 (0.43–0.55)**	**0.49 (0.4–0.6)**	**0.69 (0.58–0.83)**	**0.37 (0.32–0.42)**	**0.45 (0.35–0.59)**

Statistically significant ORs are in bold. ^*∗*^Reference SB group against which other SB categories were compared. ^†^Unadjusted ORs (95% CI) calculated using univariable logistic regression analysis. ^§^Continuous variables: age (years), MELD-Na score, and albumin (g/dL). SB, serum bicarbonate; AKI/HRS, acute kidney injury or hepatorenal syndrome; PSE, portosystemic encephalopathy; GIB, gastrointestinal bleed; SBP, spontaneous bacterial peritonitis; MELD-Na, Model of End-Stage Liver Disease-Sodium; OR, odds ratio; CI, confidence interval.

**Table 4:** Association of admission SB with cirrhosis complications during hospitalization.

Variable	AKI/HRS^†^	PSE^†^	GIB^†^	Ascites^†^	SBP^†^
SB 22–25^*∗*^	1	1	1	1	1
SB < 14	**8.49 (4.31–16.72)**	**2.22 (1.08–4.56)**	**2.19 (1.12–4.27)**	0.75 (0.39–1.43)	**2.13 (1.01–4.52)**
SB 14–17	**3.64 (2.53–5.24)**	**1.69 (1.03–2.76)**	**1.56 (1.02–2.40)**	0.97 (0.66–1.41)	1.10 (0.56–2.15)
SB 18–21	**1.99 (1.52–2.62)**	1.11 (0.73–1.68)	1.00 (0.72–1.39)	1.19 (0.90–1.56)	1.44 (0.93–2.22)
SB 26–29	0.91 (0.64–1.30)	0.97 (0.59–1.59)	**0.40 (0.25–0.65)**	1.04 (0.75–1.43)	0.70 (0.42–1.16)
SB 30–33	0.98 (0.50–1.93)	1.20 (0.50–2.90)	0.56 (0.24–1.31)	0.76 (0.39–1.50)	1.29 (0.44–3.78)
SB >33	1.52 (0.59–3.95)	2.12 (0.70–6.37)	–	1.46 (0.60–3.55)	1.92 (0.56–6.55)
Age^§^	**1.03 (1.02–1.04)**	1.01 (0.99–1.02)	0.99 (0.98–1.01)	**0.97 (0.97–0.98)**	0.99 (0.97–1.00)
Male vs female	0.94 (0.74–1.18)	1.01 (0.72–1.39)	0.90 (0.69–1.19)	**1.52 (1.21–1.92)**	1.02 (0.72–1.45)
MELD-Na^§^	**1.15 (1.13–1.17)**	**1.04 (1.02–1.06)**	0.98 (0.96–1.01)	**1.08 (1.06–1.10)**	**1.15 (1.12–1.18)**
Albumin^§^	**0.84 (0.71–0.98)**	**0.61 (0.48–0.76)**	**0.73 (0.59–0.89)**	**0.52 (0.44–0.61)**	**0.65 (0.52–0.77)**

Statistically significant ORs are in bold. ^*∗*^Reference SB group against which other SB categories were compared. ^†^Adjusted ORs (95% CI) calculated using multivariable logistic regression analysis adjusting for age, gender, MELD-Na, and serum albumin. ^§^Continuous variables: age (years), MELD-Na score, and albumin (g/dL). SB, serum bicarbonate; AKI/HRS, acute kidney injury or hepatorenal syndrome; PSE, portosystemic encephalopathy; GIB, gastrointestinal bleed; SBP, spontaneous bacterial peritonitis; MELD-Na, Model of End-Stage Liver Disease-Sodium; OR, odds ratio; CI, confidence interval.

**Table 5:** Univariable association of admission SB with adverse hospital metrics.

Variable	Hospital LOS^†^		ICU care^‡^ OR (95% CI)	Mortality^‡^ OR (95% CI)
	Mean difference (95% CI) days	*p*-value		
SB 22–25^*∗*^	0	–	1	1
SB < 14	**6.60 (3.91–9.27)**	**<0.001**	**7.27 (4.12–12.81)**	**4.09 (2.02–8.29)**
SB 14–17	**5.12 (3.58–6.66)**	**<0.001**	**2.74 (1.78–4.22)**	**3.98 (2.53–6.26)**
SB 18–21	**2.32 (1.27–3.36)**	**<0.001**	**1.69 (1.19–2.4)**	**1.63 (1.09–2.45)**
SB 26–29	**−**0.62 (**−**1.74–0.50)	0.276	0.75 (0.47–1.19)	0.68 (0.39–1.18)
SB 30–33	1.29 (**−**0.92–3.50)	0.253	1.26 (0.59–2.71)	1.54 (0.68–3.49)
SB >33	2.28 (**−**1.35-5.91)	0.217	1.36 (0.41–4.56)	0.6 (0.08–4.48)
Age^§^	**−0.06 (−0.09–0.03)**	**<0.001**	**1.01 (1.001–1.02)**	**1.02 (1.01–1.03)**
Male vs female	**−0.99 (−1.82–0.15)**	**0.020**	**0.94 (0.72–1.24)**	**0.98 (0.72–1.34)**
MELD-Na^§^	**0.30 (0.25–0.36)**	**<0.001**	**1.03 (1.01–1.04)**	**1.10 (1.08–1.12)**
Albumin^§^	**−2.91 (−3.47–2.35)**	**<0.001**	**0.59 (0.49–0.71)**	**0.34 (0.27–0.43)**

Statistically significant ORs and *p*- values are in bold. ^†^Mean difference (95% CI) in LOS calculated using general linear modeling. ^‡^ORs (95% CI) calculated using univariable logistic regression analysis. ^*∗*^Reference SB group against which other SB categories were compared. ^§^Continuous variables: age (years), MELD-Na score, and albumin (g/dL). SB, serum bicarbonate; LOS, length of stay; CI, confidence interval; ICU, intensive care unit; OR, odds ratio; MELD-Na, Model of End-Stage Liver Disease-Sodium.

**Table 6:** Association of admission SB with adverse hospital metrics.

Variable	Hospital LOS^†^		ICU care^‡^ adjusted OR (95% CI)	Mortality^‡^ adjusted OR (95% CI)
	Mean difference (95% CI) days	*p*-value		
SB 22–25^*∗*^	0	—	1	1
SB < 14	**4.07 (1.21–6.93)**	**<0.01**	**6.45 (3.50–11.89)**	1.23 (0.55–2.75)
SB 14–17	**3.14 (1.48–4.81)**	**<0.001**	**2.39 (1.52–3.77)**	**1.73 (1.04–2.87)**
SB 18–21	**1.46 (0.31–2.6)**	**0.01**	**1.72 (1.20–2.46)**	1.14 (0.73–1.77)
SB 26–29	**−**0.19 (**−**1.44–1.06)	0.77	0.80 (0.49–1.30)	0.70 (0.38–1.28)
SB 30–33	0.62 (**−**1.77–3.01)	0.61	0.98 (0.41–2.34)	1.52 (0.61–3.80)
SB >33	2.04 (**−**1.8–5.89)	0.30	1.34 (0.39–4.54)	0.49 (0.61–3.90)
Age^§^	**−0.04 (−0.08–-0.01)**	**0.02**	**1.02 (1.00–1.03)**	**1.04 (1.02–1.05)**
Male vs female	0.38 (**−**0.54–1.3)	0.42	0.87 (0.65–1.16)	0.84 (0.59–1.20)
MELD-Na^§^	**0.23 (0.17–0.3)**	**<0.001**	1.00 (0.98–1.02)	**1.11 (1.08–1.13)**
Albumin^§^	**−1.55 (−2.21–0.89)**	**<0.001**	**0.67 (0.55–0.83)**	**0.50 (0.38–0.65)**

Statistically significant ORs and *p* -values are in bold. ^†^Adjusted mean difference (95% CI) in LOS calculated using general linear modeling adjusting for age, gender, MELD-Na, and serum albumin. ^‡^Adjusted ORs (95% CI) calculated using multivariable logistic regression analysis adjusting for age, gender, MELD-Na, and serum albumin. ^*∗*^Reference SB group against which other SB categories were compared. ^§^Continuous variables: age (years), MELD-Na score, and albumin (g/dL). SB, serum bicarbonate; LOS, length of stay; CI, confidence interval; ICU, intensive care unit; OR, odds ratio; MELD-Na, Model of End-Stage Liver Disease-Sodium.

## Data Availability

The data supporting the findings of this study are available from the corresponding author upon request.

## Abbreviations

AKI: Acute kidney injury
ACLF: Acute-on-Chronic Liver Failure
aOR: Adjusted odds ratio
ANOVA: Analysis of variance
CKD: Chronic kidney disease
GIB: Gastrointestinal bleed
HRS: Hepatorenal syndrome
ICU: Intensive care unit
ICD: International classification of diseases
LOS: Length of stay
mEq/L: Milliequivalents per liter
MELD-Na: Model of End-Stage Liver Disease-Sodium
PSE: Portosystemic encephalopathy
SA: Serum albumin
SB: Serum bicarbonate
SBP: Spontaneous bacterial peritonitis.
